# TRPA1 Mediates Mechanical Currents in the Plasma Membrane of Mouse Sensory Neurons

**DOI:** 10.1371/journal.pone.0012177

**Published:** 2010-08-16

**Authors:** Daniel Vilceanu, Cheryl L. Stucky

**Affiliations:** Department of Cell Biology, Neurobiology and Anatomy, Medical College of Wisconsin, Milwaukee, Wisconsin, United States of America; Southern Illinois University School of Medicine, United States of America

## Abstract

Mechanosensitive channels serve as essential sensors for cells to interact with their environment. The identity of mechanosensitive channels that underlie somatosensory touch transduction is still a mystery. One promising mechanotransduction candidate is the Transient Receptor Potential Ankyrin 1 (TRPA1) ion channel. To determine the role of TRPA1 in the generation of mechanically-sensitive currents, we used dorsal root ganglion (DRG) neuron cultures from adult mice and applied rapid focal mechanical stimulation (indentation) to the soma membrane. Small neurons (diameter <27 µm) were studied because TRPA1 is functionally present in these neurons which largely give rise to C-fiber afferents *in vivo*. Small neurons were classified by isolectin B4 binding.

Mechanically-activated inward currents were classified into two subtypes: Slowly Adapting and Transient. First, significantly more IB4 negative neurons (84%) responded to mechanical stimulation than IB4 positive neurons (54%). Second, 89% of Slowly Adapting currents were present in IB4 negative neurons whereas only 11% were found in IB4 positive neurons. Third, Slowly Adapting currents were completely absent in IB4 negative neurons from TRPA1−/− mice. Consistent with this, Slowly Adapting currents were abolished in wild type IB4 negative neurons stimulated in the presence of a TRPA1 antagonist, HC-030031. In addition, the amplitude of Transient mechanically-activated currents in IB4 positive neurons from TRPA1−/− mice was reduced by over 60% compared to TRPA1+/+ controls; however, a similar reduction did not occur in wild-type neurons treated with HC-030031. Transfection of TRPA1 in HEK293 cells did not significantly alter the proportion or magnitude of mechanically-activated currents in HEK293 cells, indicating that TRPA1 alone is not sufficient to confer mechanical sensitivity.

These parallel genetic and pharmacological data demonstrate that TRPA1 mediates the Slowly Adapting mechanically-activated currents in small-diameter IB4 negative neurons from adult mice. The TRPA1 protein may also contribute to a complex that mediates Transient mechanically-activated currents in small IB4 positive C fiber type neurons.

## Introduction

Mechanotransduction is a fundamental mechanism by which virtually all cells from bacteria to specialized mammalian sensory neurons interact with their external environment. A myriad of physiological processes rely on the cell's ability to sense external mechanical forces, including cell division and cell volume regulation during hypo/hyperosmotic conditions [1]. Ionic channels that respond to mechanical force have been described in prokaryotic cells [2]. In microbes such as bacteria, mechanosensitive channels are directly activated by stretch of the membrane bilayer and are functionally redundant in that more than one type is expressed by the same cell [3]. In mammalian cells, a series of candidates for mechanotransduction have been proposed, including family members of TRP channels, ASIC channels, Na^+^ channels and K^+^ channels [4], [5]. However, the context of mammalian cells adds another dimension of complexity in that both the intricate extracellular matrix and intracellular cytoskeletal milieu can influence the function of mechanosensitive channels [6]. Furthermore, the functional redundancy of mechanosensitive channels is likely preserved in many, if not all eukaryotic cells, making the interpretation of ablation of a single protein challenging.

Cutaneous sensory neurons are a particularly relevant target for studying mechanotransduction because the skin is exquisitely sensitive to a wide array of mechanical stimuli ranging from delicate brush of fine hairs to painful pinch. Indeed, focal mechanical stimulation of the membrane of isolated rodent sensory neurons during patch clamp recording has revealed three general subtypes of mechanosensitive currents: Slowly Adapting, Intermediate Adapting and Rapidly Adapting [7], [8]. The Slowly Adapting current is a non-specific cation current, whereas the Rapidly Adapting current is likely carried by sodium ions [8]. Yet, the identity of the specific channel(s) underlying each current subtype is still a mystery.

One candidate for mechanotransduction in mammalian cells is the Transient Receptor Potential Ankyrin 1 (TRPA1) channel. After its characterization in a fibroblast cell line [9] and in sensory neurons [10], TRPA1 became an interesting candidate for mechanotransduction because the protein has 18 ankyrin repeats in the N terminal region. These repeats have been hypothesized to interact with the intracellular cytoskeleton and act as a spring to open TRPA1 when under mechanical stress [11], [12]. Mice deficient in TRPA1 have been shown to exhibit decreased behavioral sensitivity to intense mechanical force [13]. Recordings from skin-nerve preparations from TRPA1-deficient mice show that the firing rate of C fiber nociceptors to mechanical stimuli is decreased by 50% compared to wild type controls [14]. In parallel, acute pharmacological inhibition of TRPA1 recapitulated the genetic ablation data by showing that C fibers have markedly decreased firing rates to mechanical stimuli when a TRPA1 antagonist [15] is concomitantly applied to their receptive field [16]. Together, these findings suggest that TRPA1 plays a role in either transduction of the mechanically-gated currents or in the generation or propagation of action potentials following mechanical transduction. Of note, TRPA1 is expressed not only by sensory neurons but also by keratinocytes in the epidermis [14], [17], [18]. Thus, neither skin nerve recordings nor behavioral assays define the role of TRPA1 specifically in the sensory neuron plasma membrane.

Therefore, we set out to determine the contribution of TRPA1 to mechanically-gated currents in the sensory neuron membrane by applying rapid focal mechanical stimuli [7], [8], [19] to the somal membrane of dorsal root ganglion (DRG) neurons isolated from adult mice. Because TRPA1 has been shown to be expressed predominantly in small-diameter neurons [10], [20], many of which are C fiber nociceptors, we targeted small-diameter DRG neurons and we utilized isolectin B4 (IB4) staining to further differentiate C fiber type neurons into IB4-positive and -negative subgroups [21], [22]. Here, our parallel studies using genetic ablation and pharmacological inhibition indicate that TRPA1 mediates the Slowly Adapting mechanically-activated currents in IB4 negative C fiber type sensory neurons. Our findings demonstrate that TRPA1 contributes to the generation of mechanically-gated currents in the plasma membrane of sensory neurons.

## Methods

### Isolation of DRG neurons

Adult male mice that were wild type (Trpa1+/+, n = 21) or homozygous for deletion of TRPA1 (Trpa1−/−, n = 8) generated by Kwan and colleagues were used [13]. Mice were briefly anesthetized, killed and T10-L6 DRGs were removed from both sides. Cells were isolated as previously described [21]. Briefly, the DRGs were incubated with collagenase (1 mg/ml) followed by 0.05% trypsin (Sigma-Aldrich, St. Louis, MO) for 40 and 45 min, respectively, and mechanically dissociated into single cells using a pipette. Cells were resuspended in DMEM/Hams-F12 medium containing 10% heat-inactivated horse serum, 2 mM glutamine, 0.6% glucose, 100 units penicillin, and 100 µg/ml streptomycin (Invitrogen, Carlsbad, CA). Cells were plated onto coverslips coated with laminin (50 µg/ml; Sigma-Aldrich). No exogenous growth factors were added. Recordings were performed 12–48 hr after plating.

All procedures were approved by the Medical College of Wisconsin Animal Care and Use Committee (approval number AUA00000383) and were performed in accordance with the policies of the International Association for the Study of Pain and the National Institutes of Health.

### HEK293 cells transfection

Rat TRPA1 (Hydra Biosciences) in pcDNA5/TO vector (Invitrogen) and peGFP (enhanced Green Fluorescent Protein; Clontech, Mountain View, CA) were cotransfected overnight at a ratio of 12∶1 (rat TRPA1:peGFP) using FuGENE HD (Roche, Indianapolis, IN) in HEK293 cells. As a control, peGFP alone was transfected into HEK293 cells. After transfection the cells were treated with trypsin (0.25%, 5 min), washed, plated on glass coverslips and recorded the following day.

### Electrophysiology

Whole-cell patch-clamp recordings were performed as described previously [21]. Briefly, fire-polished glass electrodes (3**–**6 megaohms resistance) were filled with solution containing (in mM): KCl, 135; NaCl, 10; MgCl_2_, 1; EGTA, 1; NaGTP, 0.2; ATPNa_2_, 2.5; HEPES, 10; pH 7.2; osmolarity  = 290 mOsm. The recording chamber was superfused with solution containing (in mM): NaCl, 140; KCl, 5; CaCl_2_, 2; MgCl_2_, 1; HEPES, 10; glucose, 10; pH 7.4; osmolarity  = 310 mOsm. All recordings were performed at room temperature. Soma size was estimated by a calibrated eyepiece reticle. Small-diameter neurons <27 µm were primarily targeted as many of these have unmyelinated C fiber axons in vivo. Membrane voltage or current was clamped using an EPC-9 amplifier run by Pulse software (version 8.78; HEKA Electronic, Lambrecht, Germany). Neurons were included if they formed a tight seal (>1 gigaohm), had a stable resting membrane potential <−40 mV, and exhibited an action potential (AP) overshoot. Series resistance was compensated by > 60%.

### Focal Mechanical stimulation

Focal mechanical stimulation of the somal membrane was applied using a closed glass patch pipette (2**–**3 µm tip diameter) driven by a piezoelectric micromanipulator (Kleindiek MM3A-LS, Reutlingen, Germany) [8]. The pipette was positioned above the neuron at a 45° angle and its movement was controlled via NanoControl software (version 4.0; Kleindiek, Reutlingen, Germany). For each mechanical stimulus, the pipette was moved toward the soma membrane, paused for 200 ms, and then moved back to its original position. The displacement was increased by 2 µm for each consecutive stimulation until the patch seal became unstable. The displacement of the pipette ranged from 2 to 16 µm, and the actual soma membrane indentation ranged from 2 to 10 µm. An interval of 1 min was allowed between consecutive stimuli that induced mechanically-activated currents. The estimated speed of the pipette movement was 3.5 µm/ms. An inward current ≥30 pA during the mechanical stimulus was considered a response. The profile of the current was characterized as Slowly Adapting if it had a half time of inactivation over 10 ms, and Transient if it had a half time of inactivation under 10 ms. For each neuron, the current exhibiting the largest amplitude was analyzed and characterized for profile and peak amplitude. After completion of the recording, each neuron was incubated with 10 µg/ml IB4-FITC (Sigma-Aldrich, St. Louis, MO) for 10 minutes and visualized with appropriate filters as previously described [21]. A neuron was considered IB4 positive if it had a continuous green ring around the soma perimeter.

### Statistical analysis

Values are given as mean ± SEM. Differences in the current amplitudes were compared using a Student's t-test for 2 groups. Percentages of neurons responding to mechanical stimulation were compared using a Chi-square test followed by Fisher's exact test. Differences in the resting membrane potentials were compared using a Mann-Whitney U test.

## Results

### Slowly Adapting mechanical currents in wild type mice are expressed primarily by IB4 negative small neurons

**Figure 1:**
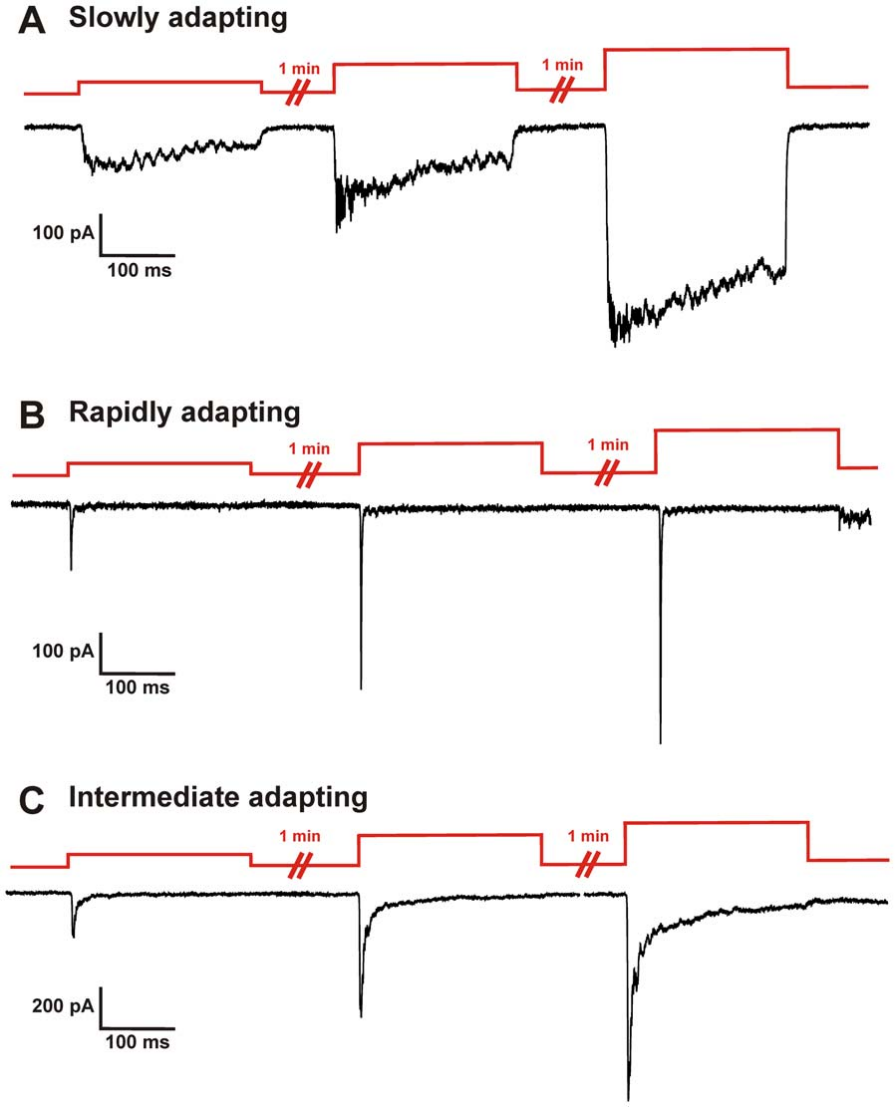
Examples of subtypes of mechanically-activated currents evoked in small-diameter DRG neurons from adult mouse. Three general subtypes of mechanical currents were observed: **A.** Slowly Adapting, **B.** Rapidly Adapting and **C.** Intermediate Adapting. Red trace indicates increasing mechanical stimuli in 2 µm increments. Responses were graded such that as the magnitude of the stimulus increased, the amplitude of the current increased proportionally. Because increasing the stimulus magnitude sometimes induced Rapidly Adapting currents to become Intermediate Adapting, these two current subtypes were combined into one group designated “Transient currents” for further analysis.

Focal mechanical stimuli to the somal membrane of adult sensory neurons induced several subtypes of inward current: Slowly Adapting, Rapidly Adapting and Intermediate Adapting, as previously described by other groups (Figure 1) [7], [8]. Because increasing the stimulus magnitude sometimes induced Rapidly Adapting currents to become Intermediate Adapting, and because Intermediate Adapting currents have the same activation and initial inactivation kinetics of Rapidly Adapting currents [23], [24] we combined Rapidly Adapting and Intermediate Adapting into one group designated “Transient currents.” Mechanically-evoked currents were characterized as Slowly Adapting (SA) if the half-time of current inactivation was >10 msec, or Transient if the half time of inactivation was <10 msec. All SA currents from wild type mice had a half-time inactivation >20 msec, whereas all Transient currents had a half-time inactivation <5 msec. Both SA and Transient mechanically-activated currents exhibited graded increases in current amplitude in response to increasing the magnitude of indentation (Figure 1). Both SA and Transient currents were completely inhibited by gadolinium (200 µM, n = 5, data not shown), a general inhibitor of stretch-activated currents, as previously shown [7], [8].

**Figure 2:**
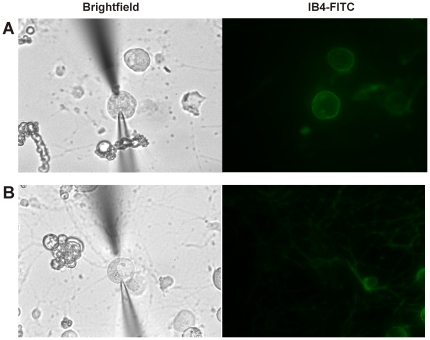
Examples of IB4 staining in small DRG neurons. **A.** A small-diameter neuron that labeled positively for IB4-FITC after patch clamp recording and maximal focal mechanical stimulation. **B.** A small-diameter neuron that was negative for IB4-FITC labeling after patch clamp recording and mechanical stimulation. Note that there are several IB4 positive neurons at the bottom right corner of the image.

**Figure 3:**
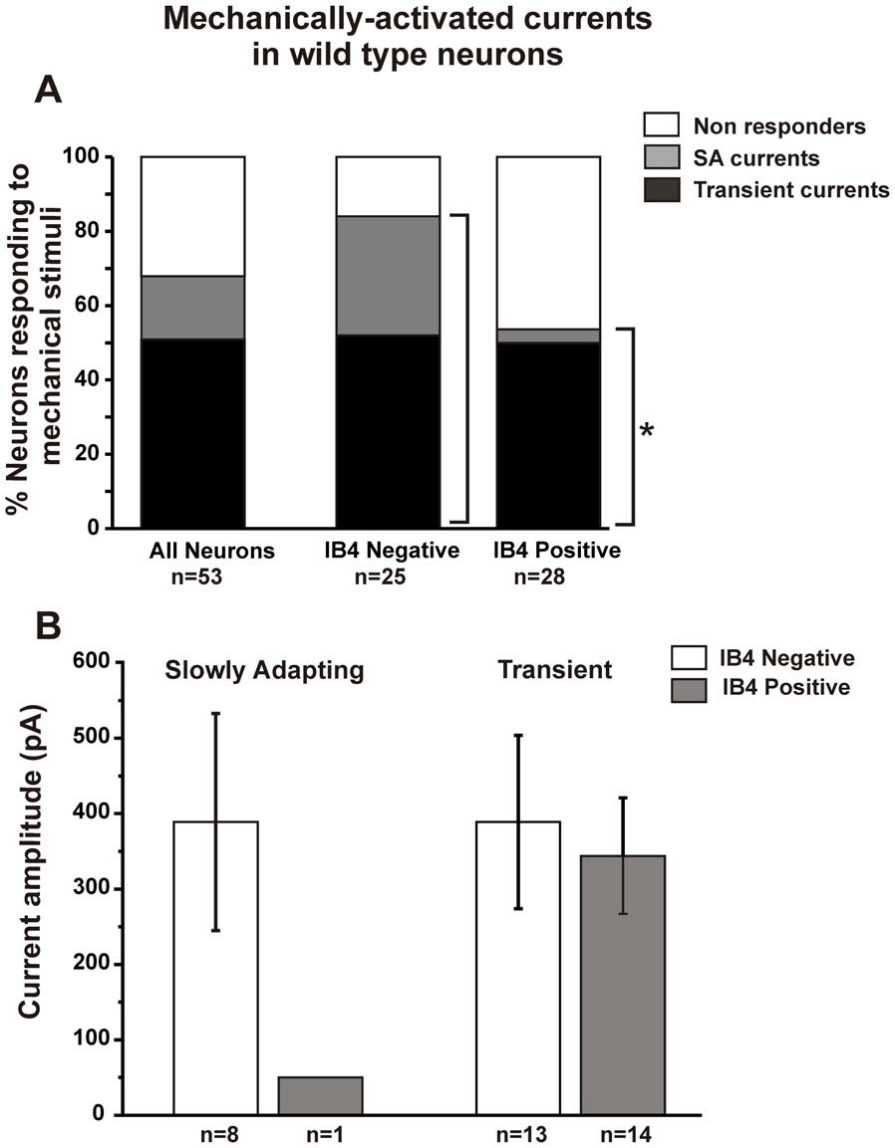
Differential expression of mechanically-evoked currents in IB4 positive and negative neurons from wild type mice. **A.** Percentage of small neurons responding to mechanical stimulation with either Slowly Adapting (SA) currents or Transient currents. More IB4 negative neurons responded to mechanical stimuli than IB4 positive neurons (* p<0.05, Fisher's exact test). SA currents were expressed predominantly by IB4 negative neurons; few were found in IB4 positive neurons. **B.** The average magnitude of the SA currents in IB4 negative neurons was much larger than the one SA current found in an IB4 positive neuron. In contrast, the magnitude of the Transient currents in IB4 positive and negative neurons was similar. Bars represent mean ± SEM.

A total of 68% (36/53) small-diameter DRG neurons from adult wild type mice exhibited a mechanically-evoked inward current whereas 32% (17/53) were mechanically-insensitive. Small neurons were then classified by their IB4 binding properties and Figure 2 shows an IB4 positive neuron (A) and an IB4 negative neuron (B) after maximum mechanical stimulation. Significantly more IB4 negative neurons (84%, 21/25) were mechanically-sensitive than IB4 positive neurons (54%, 15/28; Figure 3A; p<0.05, Fisher's exact test). Among the mechanically-sensitive neurons that expressed an SA current, the majority (89%, 8/9) were IB4 negative and only one (11%, 1/9) was IB4 positive (p<0.05; Fisher's exact test). Furthermore, the one SA current found in an IB4 positive neuron was very small in magnitude (50 pA) compared to the average magnitude of SA currents in IB4 negative neurons (∼380 pA; Figure 3B). The average magnitude of the Transient current was similar in IB4 negative and positive neurons (Figure 3B).

### Slowly Adapting mechanical currents are gone in IB4 negative neurons from TRPA1-deficient mice

**Figure 4:**
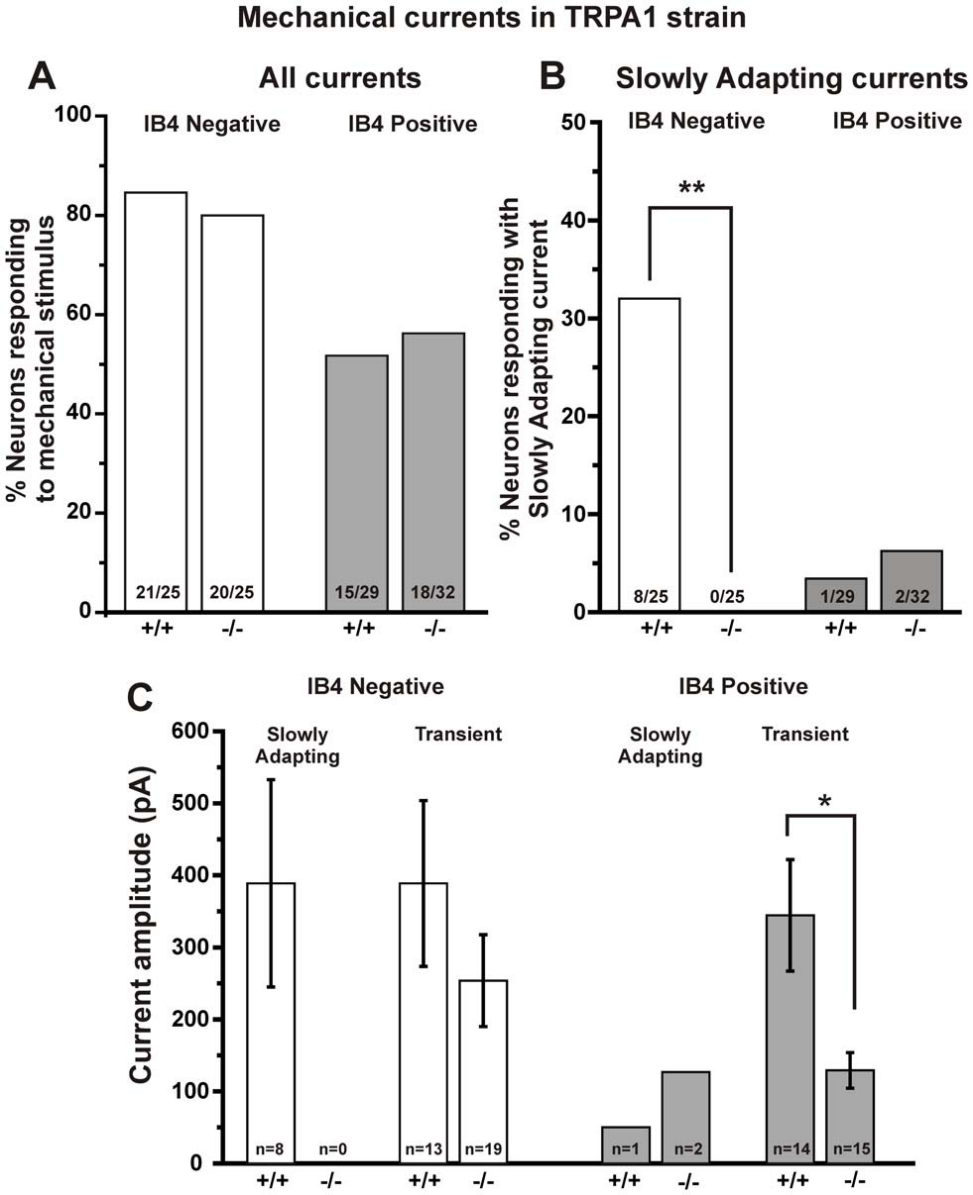
Slowly Adapting mechanical currents were gone in TRPA1−/− neurons. **A.** The total percentage of neurons expressing mechanically-evoked currents was unchanged in TRPA1−/− neurons compared to TRPA1+/+ neurons. **B.** Slowly Adapting currents were absent in IB4 negative neurons from TRPA1−/− mice compared to wild type controls (** p<0.01, Fisher's exact test). A few SA currents were found in IB4 positive neurons from both TRPA1−/− and TRPA1+/+ mice. **C.** The magnitude of the Transient currents decreased by over 60% in IB4 positive neurons from TRPA1−/− mice compared to wild type controls (* p<0.05, t-test). The magnitude of the Transient current in IB4 negative neurons was not significantly different in TRPA1−/− neurons compared to TRPA1+/+ controls. Bars represent mean ± SEM.

Next we recorded mechanically-activated currents in mice lacking TRPA1. The total percentage of small DRG neurons from TRPA1−/− mice that responded to mechanical stimulation (65%; 37/57) was not different than that in wild type controls (68%). Also, the percentage of IB4 positive or IB4 negative neurons that responded to mechanical stimuli was unaltered in TRPA1−/− mice compared to wild type controls (Figure 4A). Remarkably, however, SA currents were completely absent in the IB4 negative population in TRPA1−/− mice. Whereas 32% (8/25) of the IB4 negative neurons in wild type mice exhibited an SA mechanical current, none (0/25) of the IB4 negative neurons in TRPA1−/− mice exhibited an SA current (Figure 4B, p<0.01, Fisher's exact test). The few IB4 positive neurons that exhibited an SA current were still present in TRPA1−/− mice (Figure 4B).

Figure 4C shows the magnitude of SA and Transient current subtypes in the two subclasses of small-diameter neurons. The average peak amplitude of the Transient mechanically-activated current was not significantly different in IB4 negative neurons compared to wild type controls (Figure 4C, left). On the other hand, the amplitude of the Transient current in IB4 positive neurons from TRPA1−/− mice (122±25 pA) was only one-third as large as the Transient current in wild type controls (345±77 pA; Figure 4C right, p<0.05, t-test). Because Transient currents have been shown to be carried specifically by sodium ions [8] we investigated the action potential properties of IB4 positive neurons. Neither the amount of current necessary to generate an action potential (115±8 pA for TRPA1−/− neurons, n = 32 versus 134±15 pA for TRPA1+/+ neurons, n = 29) nor the amplitude of the action potential (99.3±2.8 mV for TRPA1−/− neurons, n = 32 versus 93.4±3 mV for TRPA1+/+ neurons, n = 29) was different in IB4 positive neurons from the two genotypes, indicating that the voltage-gated Na^+^ channels that contribute to the action potential are not functionally altered by the absence of TRPA1. Furthermore, absence of TRPA1 does not alter the IB4 binding properties of small-diameter neurons since the percentage of total small neurons that stained positively for IB4 was 53% in TRPA1−/− neurons (n = 40) and 56% in TRPA1+/+ neurons (n = 56). Also, the average soma size, capacitance and resting membrane potential were not different in IB4 positive and negative neurons recorded from TRPA1+/+ or TRPA1−/− neurons (Table S1). In addition, the percentage of small-diameter neurons that were IB4 positive versus negative after mechanical stimulation was not different than these percentages after heat or chemical stimulation or no stimulation [21], [25]. This indicates that maximal mechanical stimulation to the soma does not alter its IB4 binding properties.

### Slowly Adapting currents in wild type IB4 negative neurons are abolished by pharmacological inhibition of TRPA1

Our finding that SA currents are reduced in neurons from TRPA1−/− mice may be due to a direct contribution of TRPA1 channel activation to mechanically-gated SA currents or to alteration or downregulation of other associated proteins that normally form a structure/function complex with TRPA1. To inhibit TRPA1 channel function but leave TRPA1 protein expression in the membrane intact, we used a selective TRPA1 antagonist, HC-030031 [15], to assess mechanically-activated currents in wild type neurons. After establishing whole-cell voltage clamp mode, HC-030031 (10 µM) or vehicle (0.02% DMSO) was superfused for 2 min and mechanical stimulation was applied in the presence of the inhibitor or vehicle. To control for inter-culture and inter-animal variability, approximately 50% of neurons from a given neuronal preparation were tested with antagonist or vehicle, respectively.

**Figure 5:**
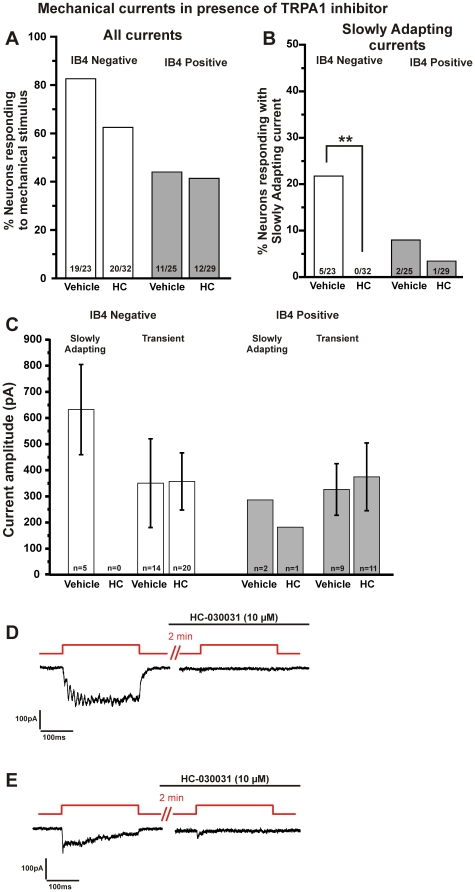
Pharmacological inhibition of TRPA1 abolishes the Slowly Adapting mechanical currents in IB4 negative neurons. **A.** The total percentage of neurons expressing mechanically-evoked currents was unaltered in neurons treated with the TRPA1 channel inhibitor, HC-030031 (10 µM), compared to those treated with vehicle (DMSO 0.02%). **B.** Slowly Adapting currents were abolished in IB4 negative neurons pretreated with HC-030031 compared to those treated with vehicle (** p<0.01, Fisher's exact test). **C.** The magnitude of the Transient mechanical currents in either IB4 positive (right) or IB4 negative (left) neurons was not altered by pretreatment with HC-030031. Bars represent mean ± SEM.**D.** Example of a neuron with a Slowly Adapting current that is subsequently completely inhibited by HC-030031. **E.** Example of a neuron with a Slowly Adapting current, where in the presence of HC-030031, a small residual Transient current remains.

Whereas 83% (19/23) IB4 negative neurons treated with vehicle responded to mechanical stimulation, 63% (20/32) of IB4 negative neurons treated with HC-030031 responded to mechanical stimuli (Figure 5A; p>0.05, Fisher's exact test). The percentage of IB4 negative neurons from vehicle-treated neurons that exhibited an SA current was 22% (5/23) and was not different than naive wild type neurons (32%, 8/25). Treatment with HC-030031 completely blocked all SA currents as none of 32 IB4 negative neurons exhibited an SA current (Figure 5B, p<0.01 when compared to the vehicle group; Fisher's exact test). As expected, HC-030031 had no effect on the magnitude of Transient currents in IB4 negative neurons (Figure 5C, left). Surprisingly, in IB4 positive neurons where the absence of TRPA1 protein reduced the magnitude of Transient currents, HC-030031 had no effect on the magnitude of the Transient currents (Figure 5C, right) as the average amplitude of the mechanically-activated Transient currents was similar for both groups (375±130 pA for HC-030031 and 326±99 pA for vehicle). Furthermore, the SA currents did not appear to be inhibited in IB4 positive neurons, as one SA current was recorded in an IB4 positive neuron in the presence of HC-030031, which is consistent with the findings in TRPA1-deficient neurons.

In a separate group of small neurons from wild type mice, an SA current was evoked and HC-030031 (10 µM) was subsequently applied for two minutes. The same magnitude focal indentation was applied again in the presence of the inhibitor. In one of three neurons, the SA current (180 pA) was completely abolished by HC-030031 (Figure 5D). In the other two neurons, a small residual Transient current remained in the presence of HC-030031. In one neuron, the initial SA current of 120 pA was reduced to a Transient of 38 pA (68% decrease; Figure 5E); the other SA current of 260 pA was reduced to a Transient of 37 pA (86% decrease; not shown).

### Expression of TRPA1 in HEK293 cells does not significantly increase mechanically-evoked currents

To determine whether expression of TRPA1 alone in heterologous cells is capable of inducing mechanically-activated currents in the plasma membrane, HEK293 cells were cotransfected with rat TRPA1 and eGFP, or transfected with eGFP alone and cells were recorded 12**–**24 hrs later. Interestingly, 19% (7/36) of HEK293 cells transfected with eGFP alone responded to focal mechanical stimulation. After tranfection with TRPA1 and eGFP, 35% (13/37) responded to mechanical stimulation but this was not significantly different than eGFP alone (p = 0.19, Fisher's exact test). There was also no difference in the peak magnitude of the mechanically-evoked currents in cells with TRPA1 and eGFP (247.8±90.9 pA; n = 13) or eGFP controls (289.6±217.1; n = 7). Of note, the cells transfected with TRPA1 and eGFP had higher average resting membrane potentials (−24.6±1.9 mV) than those transfected with eGFP alone (−49±1.9 mV; p<0.0001, Mann-Whitney U test). This suggests that the TRPA1 may have been constitutively active, resulting in a depolarized resting membrane potential.

## Discussion

Mechanotransduction in sensory neurons has come under intense investigation in recent years and is forefront in the field of sensory neurobiology [4], [26]. Sensory neurons express a wide variety of TRP channels that are thought to be in some manner, sensitive to mechanical force, including TRPA1, TRPV2, TRPV4, TRPC1, TRPC6 and TRPP2 [4], [26]. Among these, the TRPA1 channel is unique in that it has 18 ankyrin repeats in the N terminus that have been hypothesized to act as a spring when under mechanical stress [12]. Here, we investigated the role of TRPA1 in generation of mechanically-activated currents in the plasma membrane of isolated DRG neurons from adult mice using a dual approach: genetic ablation of TRPA1 and acute pharmacological inhibition of TRPA1.

The site of mechanotransduction in sensory neurons is at their peripheral terminals in the end organs (e.g. skin, muscles, viscera). However, the membrane of peripheral nerve endings is not directly accessible to functional investigation. In lieu of this, one widely used approach is to investigate the soma of isolated DRG neurons with the assumption that the soma plasma membrane mimics the peripheral terminal plasma membrane regarding the expression of the same ion channels within the same intracellular milieu. Here we applied focal mechanical stimulation to the soma of isolated DRG neurons from adult mice and reproduced the mechanically-activated currents described in the literature by other groups [7], [8], [19]. Further, we classified small-diameter DRG neurons by IB4 staining and found that IB4 negative neurons were significantly more responsive to mechanical stimulation than IB4 positive neurons. Moreover, when we stratified the mechanosensitive currents into Slowly Adapting (SA) currents and Transient currents, we found that most (89%) of the SA currents are found in IB4 negative neurons and few (only 11%) appear in IB4 positive neurons. One plausible explanation is that there is a different expression of ionic protein channels in these two subpopulations of C fiber type sensory neurons (see below). However, an intriguing alternative is that IB4 negative and positive neurons may have different plasma membrane lipid composition and thus, punctuate force or stretch stimuli may distribute differently to the ion channel proteins in the membrane that transduce external mechanical energy into membrane currents. Anecdotally, in our experience, IB4 negative neurons form tight seals in patch clamp recordings significantly faster than IB4 positive neurons (prior to staining), suggesting that IB4 negative neurons inherently possess a more dynamic plasma membrane (Vilceanu and Stucky, unpublished observations).

Mice deficient in TRPA1 have been shown to have deficits in sensing intense force applied to the hindpaw skin in behavioral assays [13]. In skin nerve preparations from these TRPA1-deficient mice, C fiber nociceptors fire 50% fewer action potentials to mechanical stimuli applied to skin receptive fields than wild type littermates [14]. Local application of a TRPA1 antagonist to skin receptive fields in wild type mice or rats induces similar decreased action potential firing [16]. Importantly, TRPA1 is expressed not only by sensory neurons but also in keratinocytes in the epidermis [14], [17], [18]. Since behavioral assays and skin nerve experiments both involve application of mechanical stimuli to keratinocytes as well as sensory nerve terminals, the site where TRPA1 physiologically contributes to mechanical activation of sensory neurons *in situ* is not clear. TRPA1 may contribute to mechanotransduction via its expression directly in the plasma membrane of the sensory terminal, or by its expression in keratinocytes that closely associate with the sensory terminal endings. Via either location, TRPA1 may ultimately contribute to mechanically-evoked action potentials in the sensory neuron, by directly contributing to transduction of the mechanical stimulus, by modulating the mechanically-activated currents, or by conveying the mechanically-evoked action potentials toward the spinal cord.

Our study indicates that TRPA1 in the sensory neuron plasma membrane participates in the generation of Slowly Adapting mechanically-activated currents. In wild type DRG neurons, SA currents are present predominantly in IB4 negative small-diameter neurons. IB4 negative neurons from TRPA1-deficient mice lack all SA currents. Similarly, pretreatment with a TRPA1 antagonist, HC-030031, inhibited all SA currents in wild type IB4 negative neurons. In some neurons with SA currents that were tested both before and after treatment with HC-030031, a very small residual Transient current remained in the presence of the inhibitor. This finding together with our finding that the total number of mechanically-sensitive neurons does not significantly decrease in the TRPA1−/− strain or in neurons pretreated with HC-030031, suggests that the SA currents may mask a Transient current that is still present in absence of TRPA1.

Our findings are consistent with evidence that SA currents are mediated by non-specific cationic channels in that TRPA1 is known to be a non-specific cationic channel [8], [10]. Interestingly however, a few SA currents were still present in IB4 positive neurons from TRPA1-deficient mice and in wild type neurons treated with the TRPA1 inhibitor. These remaining SA currents in IB4 positive neurons must be mediated by mechanically-sensitive channels other than TRPA1.

One criteria of bona fide mechanically-activated currents is that the current magnitude should be graded according to the stimulus magnitude [6]. Indeed, we show here that increasing the stimulus magnitude increases the peak current amplitude for both SA and Transient currents. In order to estimate the maximum current amplitude, we applied graded mechanical stimuli of increasing intensity until the patch clamp seal became unstable. The average amplitude of the largest mechanically-evoked current was approximately 300 pA in both IB4 positive and IB4 negative small neurons from wild type mice of the TRPA1 strain. However in neurons from TRPA1−/− mice, the amplitude of the mechanical currents decreased in IB4 positive neurons and this decrease was due to a more than 60% reduction in the amplitude of the Transient currents.

Whereas Transient currents in IB4 positive neurons were reduced in TRPA1−/− neurons, acute inhibition of TRPA1 with HC-030031 in wild type neurons did not alter the amplitude of the mechanical-activated Transient currents in IB4 positive neurons. Thus, embryonic genetic ablation of the entire TRPA1 protein and acute pharmacological inhibition of TRPA1 channel function have different effects on the Transient mechanical current. One explanation may be that the TRPA1 protein is essential to the structure-function of a mechanically-sensitive complex that mediates the Transient current phenotype/profile, and without TRPA1 protein, neurons express an attenuated Transient current. Second, the complete absence of TRPA1 may result in downregulation of the expression of other mechanically-sensitive channels essential for the Transient current. This possibility is consistent with evidence that Transient mechanically-activated currents are mediated by Na^+^ ions, whereas TRPA1 is a non-selective cation channel [8]. Third, the HC-030031 compound may fail to block mechanical activation of the TRPA1 channel mediating the Transient current in IB4 positive neurons. The site of action of HC-030031 on TRPA1 is not yet known (Magdalene Moran, personal communication), and the site(s) relevant for generation of mechanical currents may either be inaccessible to the compound or may themselves not be involved in the contribution of TRPA1 to Transient currents in IB4 positive neurons.

A definitive role of TRPA1 as a direct mechanically-gated ion channel could potentially be established through mechanical stimulation of heterologous cells expressing TRPA1. To this end, we expressed rat TRPA1 in HEK293 cells and found that although there was a trend for more mechanically-sensitive cells, the difference was not statistically significant and >50% of the TRPA1-transfected cells remained insensitive to mechanical stimuli. Furthermore, the resting membrane potential was significantly more depolarized in TRPA1-transfected cells, suggesting that the exogenous TRPA1 may have been constitutively active, resulting in a depolarized resting membrane potential. The finding that over half of the TRPA1-transfected cells were not mechanically sensitive suggests that TRPA1 alone is not sufficient to confer novel mechanical sensitivity to a cell and that associated proteins present in native neurons may be required to recapitulate mechanically-gated currents. Similarly, a recent study showed that overexpression of TRPA1 in neuroblastoma cell lines also does not confer novel mechanical sensitivity or induce any changes in endogenous mechanically-evoked currents in these heterologous cells [27].

An important consideration is that the function of an ion channel may be dependent on or heavily influenced by its native environment. The plasma membrane composition of HEK293 cells may be very different than that of DRG neurons. In native sensory neurons, TRPA1 is likely part of a macromolecular complex where it may interact intimately with other transmembrane proteins. For example, in some C fiber sensory neurons, TRPA1 may form heterotetramers with TRPV1, and the heterotetramers may have different functions than TRPA1 or TRPV1 homomers [28], [29]. Therefore, co-expression of other proteins, and possibly native sensory membrane phospholipids, may be essential to reconstitute “native” TRPA1 mechanical function in heterologous systems [27]. Furthermore, TRPA1 may be downstream to the actual mechanotransducer in the sensory membrane. For example, a different ion channel may open during mechanical stimulation, resulting in an influx of Ca^2+^ that then activates TRPA1 [30], [31].

In summary, our parallel genetic and pharmacological data indicate that TRPA1 is one of the ion channels that underlie mechanically-activated currents at the plasma membrane of sensory neurons. Specifically, TRPA1 mediates the Slowly Adapting mechanical current in IB4 negative small-diameter sensory neurons, and many of these are peptide-containing, NGF-dependent C fiber nociceptors.

## Supporting Information

Table S1 Physical and electrical properties of mechanically-activated inward currents in neurons from TRPA1+/+ and TRPA1−/− mice.
(0.04 MB DOC)

## References

[1] Stokes NR, Murray HD, Subramaniam C, Gourse RL, Louis P (2003). A role for mechanosensitive channels in survival of stationary phase: regulation of channel expression by RpoS.. Proc Natl Acad Sci U S A.

[2] Martinac B, Saimi Y, Kung C (2008). Ion channels in microbes.. Physiol Rev.

[3] Anishkin A, Kung C (2005). Microbial mechanosensation.. Curr Opin Neurobiol.

[4] Chalfie M (2009). Neurosensory mechanotransduction.. Nat Rev Mol Cell Biol.

[5] Inoue R, Jian Z, Kawarabayashi Y (2009). Mechanosensitive TRP channels in cardiovascular pathophysiology.. Pharmacol Ther.

[6] Christensen AP, Corey DP (2007). TRP channels in mechanosensation: direct or indirect activation?. Nat Rev Neurosci.

[7] Drew LJ, Wood JN, Cesare P (2002). Distinct mechanosensitive properties of capsaicin-sensitive and -insensitive sensory neurons.. J Neurosci.

[8] Hu J, Lewin GR (2006). Mechanosensitive currents in the neurites of cultured mouse sensory neurones.. J Physiol.

[9] Jaquemar D, Schenker T, Trueb B (1999). An ankyrin-like protein with transmembrane domains is specifically lost after oncogenic transformation of human fibroblasts.. J Biol Chem.

[10] Story GM, Peier AM, Reeve AJ, Eid SR, Mosbacher J (2003). ANKTM1, a TRP-like channel expressed in nociceptive neurons, is activated by cold temperatures.. Cell.

[11] Corey DP, Garcia-Anoveros J, Holt JR, Kwan KY, Lin SY (2004). TRPA1 is a candidate for the mechanosensitive transduction channel of vertebrate hair cells.. Nature.

[12] Sotomayor M, Corey DP, Schulten K (2005). In search of the hair-cell gating spring elastic properties of ankyrin and cadherin repeats.. Structure.

[13] Kwan KY, Allchorne AJ, Vollrath MA, Christensen AP, Zhang DS (2006). TRPA1 contributes to cold, mechanical, and chemical nociception but is not essential for hair-cell transduction.. Neuron.

[14] Kwan KY, Glazer JM, Corey DP, Rice FL, Stucky CL (2009). TRPA1 modulates mechanotransduction in cutaneous sensory neurons.. J Neurosci.

[15] McNamara CR, Mandel-Brehm J, Bautista DM, Siemens J, Deranian KL (2007). TRPA1 mediates formalin-induced pain.. Proc Natl Acad Sci U S A.

[16] Kerstein PC, del Camino D, Moran MM, Stucky CL (2009). Pharmacological blockade of TRPA1 inhibits mechanical firing in nociceptors.. Mol Pain.

[17] Anand U, Otto WR, Facer P, Zebda N, Selmer I (2008). TRPA1 receptor localisation in the human peripheral nervous system and functional studies in cultured human and rat sensory neurons.. Neurosci Lett.

[18] Atoyan R, Shander D, Botchkareva NV (2009). Non-neuronal expression of transient receptor potential type A1 (TRPA1) in human skin.. J Invest Dermatol.

[19] McCarter GC, Reichling DB, Levine JD (1999). Mechanical transduction by rat dorsal root ganglion neurons in vitro.. Neurosci Lett.

[20] Jordt SE, Bautista DM, Chuang HH, McKemy DD, Zygmunt PM (2004). Mustard oils and cannabinoids excite sensory nerve fibres through the TRP channel ANKTM1.. Nature.

[21] Dirajlal S, Pauers LE, Stucky CL (2003). Differential response properties of IB(4)-positive and -negative unmyelinated sensory neurons to protons and capsaicin.. J Neurophysiol.

[22] Stucky CL, Lewin GR (1999). Isolectin B(4)-positive and -negative nociceptors are functionally distinct.. J Neurosci.

[23] Lechner SG, Frenzel H, Wang R, Lewin GR (2009). Developmental waves of mechanosensitivity acquisition in sensory neuron subtypes during embryonic development.. EMBO J.

[24] McCarter GC, Levine JD (2006). Ionic basis of a mechanotransduction current in adult rat dorsal root ganglion neurons.. Mol Pain.

[25] Vilceanu D, Honore P, Hogan QH, Stucky CL (2010). Spinal nerve ligation in mouse upregulates TRPV1 heat function in injured IB4-positive nociceptors.. J Pain.

[26] Tsunozaki M, Bautista DM (2009). Mammalian somatosensory mechanotransduction.. Curr Opin Neurobiol.

[27] Rugiero F, Wood JN (2009). The mechanosensitive cell line ND-C does not express functional thermoTRP channels.. Neuropharmacology.

[28] Akopian AN, Ruparel NB, Jeske NA, Hargreaves KM (2007). Transient receptor potential TRPA1 channel desensitization in sensory neurons is agonist dependent and regulated by TRPV1-directed internalization.. J Physiol.

[29] Salas MM, Hargreaves KM, Akopian AN (2009). TRPA1-mediated responses in trigeminal sensory neurons: interaction between TRPA1 and TRPV1.. Eur J Neurosci.

[30] Zurborg S, Yurgionas B, Jira JA, Caspani O, Heppenstall PA (2007). Direct activation of the ion channel TRPA1 by Ca2+.. Nat Neurosci.

[31] Doerner JF, Gisselmann G, Hatt H, Wetzel CH (2007). Transient receptor potential channel A1 is directly gated by calcium ions.. J Biol Chem.

