# Supplementary material for: TRPA1 Mediates Mechanical Currents in the Plasma Membrane of Mouse Sensory Neurons
Source: PLoS One. 2010 Aug 16;5(8):e12177. doi: 10.1371/journal.pone.0012177 (PMC2922334; doi:10.1371/journal.pone.0012177)
Supplement: Table S1 — Physical and electrical properties of mechanically-activated inward currents in neurons from TRPA1+/+ and TRPA1−/− mice. (0.04 MB DOC) [file pone.0012177.s001.doc]

**Table 1: Physical and electrical properties of mechanically-activated inward**

**currents in neurons from TRPA1+/+ and TRPA1-/- mice**

|  | N | Diameter (µm) | Capacitance (pF) | Membrane Potential (mV) |
| --- | --- | --- | --- | --- |
| **TRPA1+/+ IB4 Neg** |  |  |  |  |
| Slowly Adapting | 8 | 21.7±0.9 | 17.6±0.9 | -52.5±1.6 |
| Transient | 13 | 21.5±0.4 | 17.2±0.7 | -50.5±2.0 |
| Non-Responders | 4 | 23.0±0.8 | 19.8±1.9 | -54.5±1.9 |
| **TRPA1+/+ IB4 Pos** |  |  |  |  |
| Slowly Adapting | 1 | 23.6 | 19.32 | -43 |
| Transient | 14 | 21±0.5 | 17.1±0.7 | -50.6±1.9 |
| Non-Responders | 13 | 21.9±0.5 | 17.7±0.9 | -47.2±1.1 |
| **TRPA1-/- IB4 Neg** |  |  |  |  |
| Slowly Adapting | 0 | --- | --- | --- |
| Transient | 20 | 21.6±0.4 | 17.7±0.6 | -50.1±1.6 |
| Non Responders | 5 | 23.0±0.5 | 17.6±1.3 | -49.2±2.2 |
| **TRPA1-/- IB4 Pos** |  |  |  |  |
| Slowly Adapting | 2 | 23.2 | 21 | -50 |
| Transient | 15 | 22.5±0.4 | 18.7±0.8 | -50.2±1.4 |
| Non-Responders | 14 | 21.1±0.6 | 16.8±1.3 | -48.6±1.3 |
